# Pharyngeal metastasis following living-donor liver transplantation for hepatocellular carcinoma: a case report and literature review

**DOI:** 10.1186/s12957-020-01873-0

**Published:** 2020-05-28

**Authors:** Taiji Tohyama, Katsunori Sakamoto, Kei Tamura, Taro Nakamura, Jota Watanabe, Hiroyuki Wakisaka, Yasutsugu Takada

**Affiliations:** 1 Department of Hepato-Biliary-Pancreatic and Breast Surgery, Ehime University Graduate School of Medicine, Shitsukawa, Toon, Ehime, 791-0295 Japan; 2 Department of Surgery, Kurashiki Medical Center, Bakuro-cho, Kurashiki, Okayama, 710-8522 Japan; 3 Laboratory of Head and Neck Surgery, Ehime Prefectural University of Health Sciences, 543, Takoda, Tobe-cho, Iyo-gun, Ehime, 791-2101 Japan

**Keywords:** Hepatocellular carcinoma, Pharyngeal metastasis, Nasopharynx, Liver transplantation, Vertebral venous plexus

## Abstract

**Background:**

The most common sites of recurrence after liver transplantation for hepatocellular carcinoma (HCC) have been reported to be the liver, lung, bone, and adrenal glands, but there have also been many reports of cases of multiple recurrence. The prognosis after recurrence is poor, with reported median survival after recurrence of HCC ranging from 9 to 19 months. Here, we report a case of long-term survival after recurrence of pharyngeal metastasis following living-donor liver transplantation (LDLT) for HCC within the Milan criteria, by resection of the metastatic region and cervical lymph node dissection.

**Case presentation:**

A 47-year-old man with a Model End-stage Liver Disease (MELD) score of 11 underwent LDLT for HCC within the Milan criteria for liver cirrhosis associated with hepatitis B virus infection, with his 48-year-old elder brother as the living donor. One year and 10 months after liver transplantation, he visited a nearby hospital with a chief complaint of discomfort on swallowing. A pedunculated polyp was found in the hypopharynx, and biopsy revealed HCC metastasis. We performed pharyngeal polypectomy. Two years later, cervical lymph node metastasis appeared, and neck lymph node dissection was performed. Although recurrence subsequently occurred three times in the grafted liver, the patient is still alive 12 years and 10 months after recurrence of pharyngeal metastasis. He is now a tumor-free outpatient taking sorafenib.

**Conclusion:**

It is necessary to recognize that the nasopharyngeal region is a potential site of HCC metastasis. Prognostic improvement can be expected with close follow-up, early detection, and multidisciplinary treatment, including radical resection.

## Background

In 1996, Mazzaferro et al. reported that when liver transplantation for hepatocellular carcinoma (HCC) was restricted to patients with early-stage disease (defined as a single tumor less than 5 cm in diameter, or up to three tumors less than 3 cm in diameter, without vascular invasion or extrahepatic metastasis), the 4-year survival rate was 75%, similar to survival rates for patients with non-HCC liver disease [1]. These criteria, which were soon termed the “Milan criteria,” became the worldwide benchmark for liver transplantation criteria for HCC. The Milan criteria, limiting the indications for liver transplantation for HCC, have reduced the postoperative recurrence rate of HCC. However, HCC still recurs in 1.6–13% of recipients within the Milan criteria after liver transplantation [1–6]. There have been many reports of cases with multiple instances of recurrence due to systemic recurrence caused by extra-hepatic circulating tumor cells. The prognosis after recurrence of HCC is poor, with reported median survival ranging from 9 to 19 months in major transplant centers in both Western and Eastern countries [4, 7–12]. However, if the recurrence remains local, such as within the liver, lung, or adrenal gland, surgical resection may improve prognosis, and cases of long-term survival have been reported [4, 6–8, 10, 11, 13]. Here, we report a case of long-term survival after resection of the pharyngeal metastasis due to HCC recurrence after living-donor liver transplantation (LDLT).

## Case presentation

**Fig. 1:**
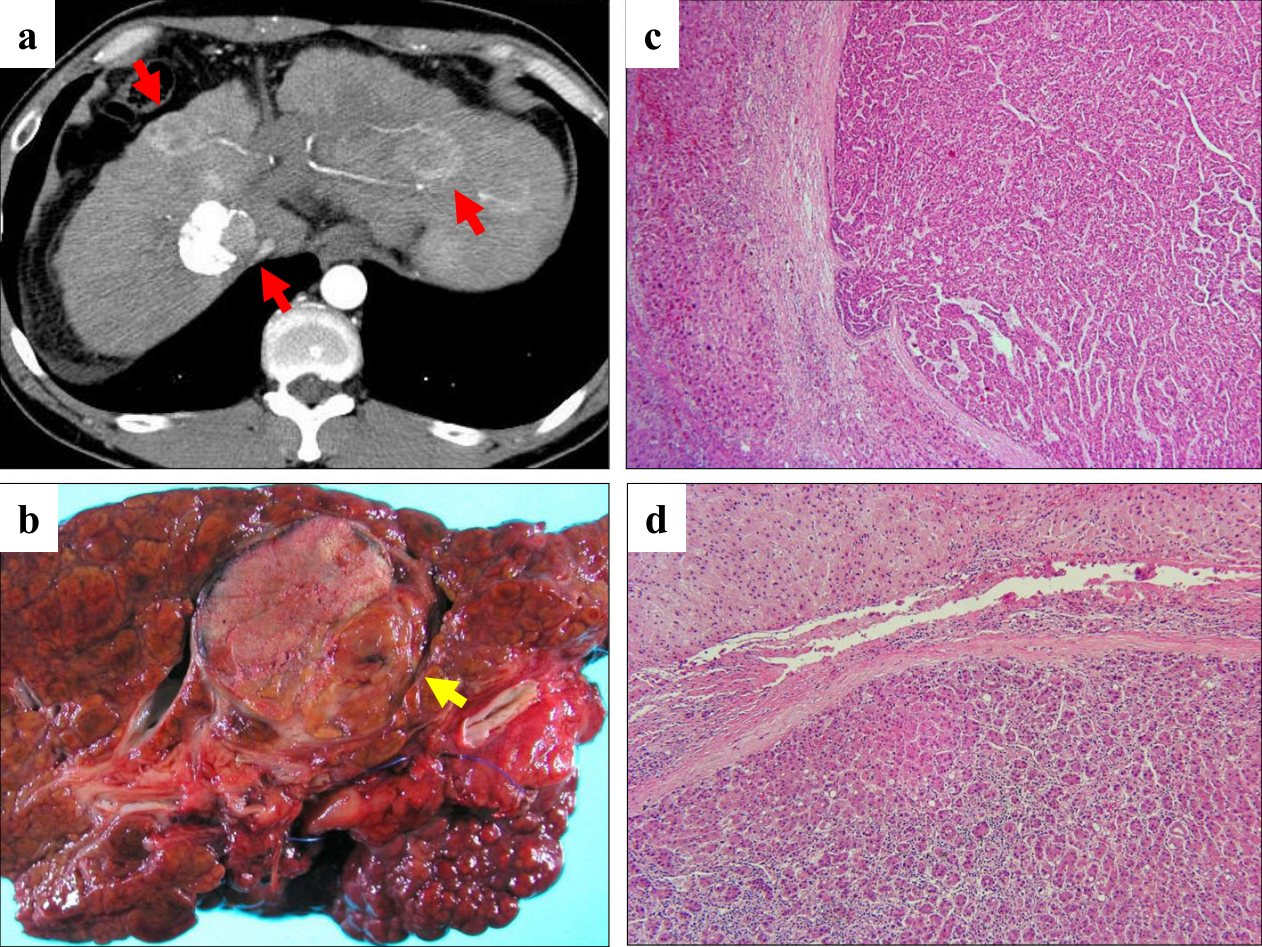
Computed tomography (CT) images before living-donor liver transplantation and histopathological findings of the resected native liver. **a** Preoperative abdominal CT showing liver cirrhosis with three hypervascular nodules that were 20, 25, and 28 mm in diameter. Embolism deposits due to transarterial chemoembolization (TACE) were observed in one of the three S1 tumors. **b** Macroscopic examination of the resected liver revealed the presence of a tumor 28 mm in diameter with partial necrosis due to preoperative TACE in segment 1, but no tumors were observed in other areas, only regenerated nodules. **c**, **d** Microscopic view of moderately differentiated hepatocellular carcinoma (HCC) with necrotic changes in segment 1 and multiple well-differentiated HCC lesions with trabecular and pseudoglandular structures measuring a few millimeters in diameter

**Fig. 2:**
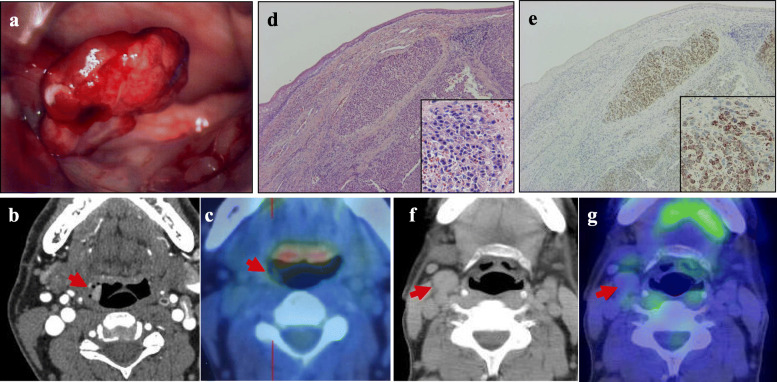
Pharyngeal metastasis and cervical lymph node metastasis following living-donor liver transplantation for hepatocellular carcinoma (HCC). **a** Macroscopic findings of a pedunculated polyp-shaped pharyngeal metastatic lesion. **b** Cervical computed tomography (CT) revealing a pharyngeal polyp on the right side of the epiglottis. **c** Faint fluorodeoxyglucose (FDG) accumulation consistent with the pharyngeal polyp as observed using FDG positron emission tomography (PET)-CT. **d** Histological examination showing polygonal dysplastic epithelial cells with a trabecular or pseudotubular structure that had proliferated under the mucous membrane covered with the squamous epithelium. **e** These tumor cells stained positively for anti-hepatocyte-specific antigen and were diagnosed as indicative of HCC metastasis. **f** Cervical CT showing regional neck lymph node swelling at 2 years after pharyngeal polypectomy. **g** PET-CT scan indicating that there was almost no FDG accumulation in the enlarged cervical lymph node

**Fig. 3:**
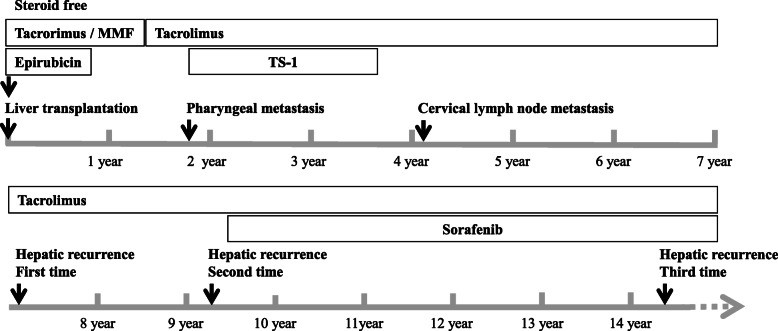
Clinical course of patient with pharyngeal metastasis after living-donor liver transplantation (LDLT) for hepatocellular carcinoma (HCC). Tacrolimus and mycophenolate mofetil were used as posttransplant immunosuppressive agents, with no steroid administration. Epirubicin was administered during surgery. Pharyngeal metastasis occurred at 1 year and 10 months after LDLT. In addition, at 2 years after pharyngeal polypectomy, recurrence of HCC was detected in the regional neck lymph nodes. Recurrence subsequently occurred three times in the grafted liver, and local treatment with transcatheter arterial chemoembolization and radiofrequency ablation therapy were performed. The patient took TS-1 orally after pharyngeal polypectomy for approximately 1 year and 6 months, and oral administration of sorafenib was started after the second liver graft recurrence

A 47-year-old man with a Model End-stage Liver Disease (MELD) score of 11 and liver cirrhosis associated with hepatitis B virus (HBV) infection underwent LDLT for HCC, with his 48-year-old elder brother as the living donor, in November 2004. He had received transarterial chemoembolization (TACE) 5 months before liver transplantation and was assessed as having a partial response according to the modified Response Evaluation Criteria in Solid Tumors (mRECIST) [14]. The patient had no history of other diseases, and there were also no diseases of note in his family history. The right lobe of the donor was harvested and weighed 710 g (graft-to-recipient weight ratio, 1.09). The donor and recipient had the same blood type. Preoperative abdominal computed tomography (CT) revealed liver cirrhosis with three hypervascular nodules, diagnosed as HCC, which were 2.0, 2.5, and 2.8 cm in diameter and met the Milan criteria (Fig. 1a). Preoperative tumor marker levels were as follows: α-fetoprotein (AFP), 5 ng/ml; and des-ɤ-carboxy prothrombin (DCP), 327 mAU/ml. Pathological examination of the resected liver revealed one moderately differentiated HCC with necrotic change in a 2.4-cm area, and multiple well-differentiated HCC lesions measuring a few millimeters that appeared to indicate multicentric occurrence. Microvascular invasion was not seen (Fig. 1b, c, d). We used tacrolimus and mycophenolate mofetil (MMF) as posttransplant immunosuppressive agents; no steroids were employed. The early postoperative course was uneventful, and the patient was discharged 74 days postoperatively. He received epirubicin at a dose of 10 mg/m^2^ during surgery, but did not receive postoperative chemotherapy. He was followed up by abdominal ultrasonography (US) every 3 months and by abdominal CT at 1, 3, 6, and 12 months after surgery, and every 6 months thereafter. The AFP and DCP levels were checked monthly. Lamivudine was administered to control HBV virus, and HBV DNA was consistently negative after transplantation. In September 2006, 22 months after LDLT, he visited a nearby hospital with a chief complaint of discomfort on swallowing. A pedunculated polyp was found in the hypopharynx (Fig. 2a), and biopsy revealed HCC metastasis. Cervical CT revealed a pharyngeal polyp on the right side of the epiglottis; also, faint fluorodeoxyglucose (FDG) accumulation was recognized, consistent with a pharyngeal polyp on FDG positron emission tomography (PET)-CT examination (Fig. 2b, c). Polypectomy was performed on October 2006. Histological examination revealed that the tumor cells were positive for anti-hepatocyte antigen staining and a diagnosis of metastasis of HCC was made (Fig. 2d, e). He was administered tegafur/gimeracil/oteracil (TS-1) after polypectomy. Two years after pharyngeal polypectomy, recurrence in neck lymph nodes was detected (Fig. 2f, g), and neck lymph node dissection was performed. Recurrence subsequently occurred three times in the graft liver, and local treatment with TACE and radiofrequency ablation therapy was performed. The patient was started on oral sorafenib 3 years ago, is still alive 12 years and 10 months after recurrence of pharyngeal metastasis, and is now a tumor-free outpatient in good health continuing to take a low dose of sorafenib (Fig. 3).

## Discussion

Liver transplantation is the only curative treatment for HCC associated with severe liver cirrhosis [15–17]. The critical shortage of deceased donor organs has led to the rapid development of LDLT in Eastern countries, including Japan, Korea, and China. And in contrast to deceased donor liver transplantation, recipient selection for LDLT is not limited by organ allocation systems. Subsequently, many centers have developed expanded center-specific criteria with acceptable results, including the University of California San Francisco criteria, Extended Toronto criteria, and Kyoto criteria and others [18–24].

A major issue with liver transplantation for HCC is that recurrence of HCC is associated with a high risk of death within a short time. The prognosis after recurrence is very poor, with reported median survival times ranging from 9 to 19 months [4, 7–11]. No effective postoperative adjuvant chemotherapy for HCC after liver transplantation has been established. To date, the best outcomes have been achieved by limiting the indications for transplantation according to tumor status. The Milan criteria have become the global standard guidelines for liver transplantation for HCC. Nevertheless, rates of recurrence of HCC within the Milan criteria after liver transplantation have been reported to be 1.6–13% [1–6].

The most commonly reported initial sites of local recurrence after liver transplantation for HCC are the grafted liver (15%), lung (20–30%), bone (20–25%), abdominal lymph node (15%), and adrenal glands (5%) [2–4, 10, 25]. However, the rate of initial recurrence in multiple sites was reported to be approximately 40% when systemic recurrence is promoted by circulating tumor cells; solitary recurrence is very rare, except at those sites [2, 9, 10, 25]. There are seven published cases of pharyngeal metastasis from HCC, which are summarized along with our case in Table 1 [26–32]. The reports all describe male patients with a median age of 70 years (range 49–71 years). Four cases of synchronous metastasis and four cases of metachronous metastasis have been reported. The time between the first treatment and pharyngeal metastasis was 17–58 months in cases of metachronous metastasis. Five patients, including our case, underwent surgery for pharyngeal metastases. The prognosis for all cases except ours was poor; four died within 1 year, two underwent difficult curative treatment, and one experienced multiple recurrences of pharyngeal metastasis at 1 year after surgery [28–32]. Our report is the second to describe solitary recurrence in the pharyngeal region after liver transplantation and includes the longest surviving case of HCC with pharyngeal metastasis.

**Table 1:** Characteristics of patients with pharyngeal metastasis from hepatocellular carcinoma

Case	Author, year (reference)	Age	Sex	Treatment for primary HCC	Maximum size of primary HCC (cm)	Number of primary HCC	Histopathology of primary HCC	Interval from primary HCC to pharyngeal metastasis	Treatment for metastasis	Outcome
1	Ciriza,1996 [26]	71	M	None	2.4	1	Unknown	3 years	Operation	Died after 8 months
2	Llanes, 1996 [27]	71	M	None	8	1	Unknown	Synchronous	Operation	Died after 10 months
3	Oida, 2005 [28]	59	M	Hepatectomy	3	1	Moderate	Synchronous	Operation and radiation	Died after 8 months
4	Nagano, 2008 [29]	73	M	Hepatectomy, TAE, RFA	3	1	Unknown	4 years 10 months	Operation	Alive for 1 year with multiple instances of recurrence in the remnant liver
5	Kattepur, 2014 [30]	70	M	None	1.9	1	Unknown	Synchronous	None	–
6	Guo, 2015 [31]	50	M	TAE	Large, diffuse	1	Unknown	Synchronous	Radiation	–
7	Lou, 2019 [32]	45	M	Liver transplantation	4	1	Unknown	1 year 5 months	Radiation	Died after 3 months
8	Present case	49	M	Liver transplantation	2.4	Multiple	Well–moderate	1 year 10 months	Operation	Alive for 12 years 10 months

*HCC* hepatocellular carcinoma, *M* male, *TAE* transarterial embolization, *RFA* radiofrequency ablation therapy

The mechanism of metastasis to the pharyngeal region is controversial, although it has been speculated that there are two main pathways: tumor cells may circulate through the vena cava and be distributed to the pharyngeal region via the arterial systemic circulation, or may reach the head and neck by bypassing the lungs, possibly through the portal–vertebral venous plexus (Batson’s theory) [33]. The vertebral venous plexus consists of the internal vertebral venous system distributed around the spinal canal, the external vertebral venous plexus distributed in front of the vertebral body, and the vertebral vein that anastomoses both of those sites. This plexus communicates with the intercostal vein and the azygos vein in each site. In the head and neck region, the plexus communicates with the pterygium venous plexus, cavernous venous plexus, and pharyngeal venous plexus around the nasal and paranasal sinuses [34]. As there is no venous valve in the vertebral vein, the blood is thought to easily flow backward when the intrathoracic pressure or abdominal pressure rises [33]. HCC metastasis through the portal vein is considered to be common, such that tumor cells that have entered the vertebral vein plexus from the portal vein flow back to the pharyngeal venous plexus due to an increase of intraperitoneal pressure, and metastasize to the pharynx [33–35]. In this case, metastasis first occurred as a pharyngeal polyp, and recurrence occurred in the regional lymph node on the same side 2 years later. During this time, metastasis to other organs, including the lungs, did not occur; thus, it was strongly suspected that it had spread to the pharynx via the vertebral vein plexus of Baston. In 2005, Oida et al. reviewed 10 cases of HCC with pharyngeal metastasis (non-English articles included) [28]. In addition, Hou et al. reported 30 cases of HCC metastasis in the gingival region [36]. Collectively, these cases imply that oral cavity and nasopharyngeal metastasis via the portal-vertebral venous plexus represent a primary HCC metastatic pathway.

The Milan criteria are based on the size and number of HCCs. In addition to tumor size and number, the grade of histological differentiation, microvascular invasion, and underestimation of HCC burden based on preoperative imaging are reported to be associated with recurrence [2, 4, 37, 38]. Furthermore, there is an increased desire for further information on tumor biology and surrogates of tumor biology when determining transplant suitability. Consequently, some transplantation centers include tumor markers in their patient evaluations, such as AFP and DCP, the levels of which are correlated with HCC recurrence rate [2, 20, 39]. The HCC response to pretransplant locoregional therapy is an important surrogate marker for survival, as well as an indicator of tumor biology. Some authors reported that the 5-year overall survival rate and recurrence rate were significantly associated with pretransplant treatment for HCC [40, 41]. In this case, pretransplant TACE for HCC resulted in a partial response, small multiple well-differentiated HCCs that appeared to represent multicentric occurrence were observed during pathological examination, and the DCP level was high preoperatively. Therefore, this case fell within the Milan criteria based on preoperative imaging, but the risk of recurrence was considered high.

Even if the vertebral vein system develops as a collateral pathway, this does not manifest in any specific symptom, and its recognition as a transportal-vertebral metastatic pathway remains poor. In addition, metastasis in pharyngeal regions is difficult to detect because such regions represent the border between the head and chest on CT examinations. Metastasis in pharyngeal regions may be missed by common follow-up imaging procedures, such as US and CT, such that recurrence may be discovered only after metastasis to other sites. Early detection is the only factor associated with effective treatment after recurrence. Careful follow-up involving the analysis of tumor markers and imaging analysis of frequent metastatic recurrence sites, including the pharyngeal and cervical regions, are important, especially in high-risk patients.

Over the last decade, we have significantly improved our understanding of the molecular landscape of HCC [42]. Numerous studies have investigated the utility of molecular biomarkers, such as DNA alterations, to predict outcomes in patients following liver transplantation for HCC [42]. Circulating miRNA has emerged as one of the most attractive tools for the early diagnosis of cancers [42–44]. Some authors have reported that miRNA markers were useful predictors of HCC recurrence after liver transplantation [42, 45–47]. In the near future, it is expected that the understanding of tumor biology will advance through further analysis of these molecular mechanisms, facilitating individual risk stratification for HCC patients who will benefit from liver transplantation [42].

The concept of oligorecurrence, introduced by Hellman and Weichselbaum in 1995, suggests that survival is improved by aggressive local treatment depending on the number and location of recurrent tumors [48]. Extrahepatic recurrence of HCC often occurs at local sites after liver resection, and survival can be prolonged by surgical resection in such cases [49, 50]. Even after liver transplantation, prolongation of survival can be expected if radical resection is performed for local recurrence [4, 6, 8, 10, 11, 13]. Although there are no strict surgical indications for HCC recurrence after liver transplantation, the recurrence time following liver transplantation is 12–24 months or more; the prognosis of surgery for recurrence is good [4, 10, 13]. Other prognostic factors have been revealed, such as an AFP level of 100 ng/ml or greater, bone metastasis, three or more tumors, and tumor size at the time of recurrence [8, 10, 11, 13]. About 10–23% of patients with recurrence survive for a long time after surgical therapy [6, 8, 51]. The median survival of unresectable cases was reported to be 5–15 months, while that of resectable cases with local recurrence was reported to be 28–65 months; cases of long-term survival after resection of the grafted liver, lung, and adrenal metastasis have also been reported [52–55].

The factor responsible for the favorable clinical course in our case is thought to be that surgery could be performed easily to treat pharyngeal recurrence because of the pedunculated polyp-like shape at the site of recurrence, and radical resection of subsequent lymph node recurrence was possible. In addition, we used a steroid-free postoperative immunosuppressant regimen. After recurrence of HCC, TS-1 and sorafenib were used as antitumor agents. Several studies have indicated that immunosuppressant therapy including steroid can impact HCC recurrence after liver transplantation [56]. The mammalian target of rapamycin (mTOR) inhibitors sirolimus and everolimus have been reported to be effective as immunosuppressants after liver transplantation for HCC [57, 58]. However, data on the utility of mTOR inhibitors for treating HCC recurrence after liver transplantation remain scarce [48].

Recently, new anticancer and molecular targeted drugs, such as sorafenib, regorafenib, lenvatinib, ramucirumab, and nivolumab, have been clinically applied to treat HCC. The efficacy of these drugs for treating recurrence after liver transplantation for HCC has been studied; several reports indicated prolongation of survival, but there is no established therapeutic regimen and there is some concern regarding adverse effects on liver function [59–62]. Clinical trials of chemotherapy for patients with HCC recurrence and/or postoperative adjuvant chemotherapy for patients at high risk of recurrence after liver transplantation are required [63].

## Conclusion

In summary, we have reported a rare case of pharyngeal metastasis following LDLT for HCC. The cervical region must be recognized as a primary site of extrahepatic metastasis of HCC via the portal–vertebral venous plexus. Even with recurrence in the pharyngeal region, patients can achieve long-term survival, as in the present case. Early diagnosis of recurrence via careful periodic follow-up examinations of the cervical to nasopharyngeal region, together with frequent sites of metastatic recurrence such as the lungs, grafted liver, adrenal glands, bone, and abdominal lymph nodes, is required. At present, there is no established immunotherapy or adjuvant chemotherapy after liver transplantation that suppresses HCC recurrence. In addition, the effectiveness of surgical treatment for the oligo-recurrence of HCC after liver transplantation has been reported, but there is no effective treatment for multiple recurrences. Multidisciplinary treatment with radical resection and various subsequent treatments, including immunosuppressive treatment and anti-tumor therapy, are also needed according to the patient’s condition.

## Data Availability

All data generated or analyzed are included in this published article.

## Abbreviations

HCC: Hepatocellular carcinoma
LDLT: Living-donor liver transplantation
MELD: Model End-stage Liver Disease
HBV: Hepatitis B virus
CT: Computed tomography
AFP: α-Fetoprotein
DCP: Des-ɤ-carboxy prothrombin
MMF: Mycophenolate mofetil
US: Abdominal ultrasonography
FDG: Fluorodeoxyglucose
PET: Positron emission tomography
TS-1: Tegafur/gimeracil/oteracil
mTOR: Mammalian target of rapamycin
